# Supplementary material for: Chromothripsis during telomere crisis is independent of NHEJ, and consistent with a replicative origin
Source: Genome Res. 2019 May;29(5):737–49. doi: 10.1101/gr.240705.118 (PMC6499312; doi:10.1101/gr.240705.118)
Supplement: Supplemental Material [file supp_gr.240705.118_Supplemental_file_1.zip › contigs/annotated_contigs/DB73/contig.2.DB73_length_433_mean_cov_4.84988452656.docx]

**DB73_length_433_mean_cov_4.84988452656**

```
ATTTGTTACCACTGCCCTAATAACCGTGCCCTGACCTCCAGCTGCTAGAGAGAGGATGTCCCCCTAGTAAAGCCAAGCAGGAATTGAAG
 >chr19:54683354-54683468 - E=4e-57
GTTTTTCTAAATCTGCTCGGTCC|TC|CCAATAAAGTGTTGCACAAAGGCATCGTGTCCGTGCAGGTATCTGGGTTATAAACGGTGGGA
                        >chr19:54676810-54677131 - E=9e-167
AGGACTTAGTCCACCAAGTCCCAGGGTGAGGTACAGCCCCCCCGCCCAGCCCAGGAACCAAACTGTCAGGCCCGGGGCACCACGGGGAC

TTCAGCTCCCAGGAGACCTTTCGCATCAGCGGCCCTGAGAAACCACAGGAAGTGTACCTTACTCCCTCCGGGCCACCTGCTGGCCAGGT

ACACACCTGCCCCTGGCCCCTCCCCTACCTGGGGCAGTGTCTGCCTGGTGGCCACCAGACACAGCCCAGCCTGGGCCAT
```
